# Probiotic *Bacillus subtilis*, but Not a *Lactobacillus* spp., Ameliorates Cognitive Impairment in a Mouse Model of LPS and Zidovudine-Induced Neuroinflammation

**DOI:** 10.3390/brainsci16030340

**Published:** 2026-03-21

**Authors:** Olga Murgina, Ksenia Stafeeva, Sofya Karaulova, Alena Vostrikova, Sofya Kononova, Daria Chursina, Svetlana Pozdeeva, Anastasia Makogonova, Inna Burakova, Svetlana Pogorelova, Polina Morozova, Yulia Smirnova, Mikhail Syromyatnikov, Viktor Shutikov, Evgeny Mikhailov, Artem Gureev

**Affiliations:** 1 Department of Genetics, Cytology and Bioengineering, Voronezh State University, Voronezh 394018, Russia; karandeevaolha@yandex.ru (O.M.); kseniyastafeeva@gmail.com (K.S.); karaulova.sofya@inbox.ru (S.K.); a.reyk@bk.ru (A.V.); sophiakononova@yandex.ru (S.K.); k4slin@yandex.ru (D.C.); spozdeeva32@gmail.com (S.P.); anastasia.kokina@mail.ru (A.M.); syromyatnikov@bio.vsu.ru (M.S.); 2 Laboratory of Metagenomics and Food Biotechnology, Voronezh State University of Engineering Technologies, Voronezh 394036, Russia; vitkalovai@inbox.ru (I.B.); zubkowa.sweta@gmail.com (S.P.); ms.cloud00.00@mail.ru (P.M.); dyd16@mail.ru (Y.S.); 3 Laboratory of Innovative Preparations of Recombinant Proteomics, All-Russian Veterinary Research Institute of Pathology, Pharmacology and Therapy, Voronezh 394087, Russia; shutikov.02@yandex.ru; 4 Department of Experimental Pharmacology and Functioning of Living Systems, All-Russian Veterinary Research Institute of Pathology, Pharmacology and Therapy, Voronezh 394087, Russia; voronezh81@rambler.ru

**Keywords:** gut–brain axis, cognitive impairment, lipopolysaccharide, zidovudine, probiotic, *Bacillus subtilis*, *Lactobacillus* spp., microbiome, neuroinflammation, mitochondrial dysfunction

## Abstract

**Highlights:**

**What are the main findings?**

• LPS + ZDV cause gut–brain axis dysfunction in mice.
• *B. subtilis* probiotic improves cognition and gut health.

**What are the implications of the main findings?**

• It reduces brain mitochondrial damage and systemic inflammation.
• *Lactobacillus* spp. Mix is ineffective and harmful to intestines.

**Abstract:**

**Background/Objectives:** The gut–brain axis is increasingly recognized as a critical modulator of cognitive function. This study investigated the neurotoxic effects of combined exposure to bacterial lipopolysaccharide (LPS) and the antiretroviral drug zidovudine (ZDV) in a mouse model, and evaluated the protective potential of two probiotic interventions: Bacillus subtilis and a mixture of lactobacilli. **Methods:** Cognitive function was assessed using the Morris water maze (MWM). Gut microbiota composition was analyzed by 16S rRNA sequencing, and intestinal morphology was examined histologically. Gene expression of neuroinflammatory markers and mitophagy-related genes in brain tissue was quantified by RT-PCR. Plasma levels of cell-free mitochondrial DNA (cf-mtDNA) were measured as a marker of mitochondrial damage. **Results:** Combined LPS + ZDV exposure induced systemic inflammation, impaired spatial memory, damaged the intestinal mucosa, and caused dysbiosis characterized by an increase in pro-inflammatory *Muribaculaceae*. In the brain, LPS + ZDV significantly upregulated *Tnfa* expression, confirming neuroinflammation. *Bacillus subtilis* administration prevented cognitive deficits, maintained *Tnfa* at control levels, and significantly reduced *Il1b* and *Il6* expression compared to the LPS + ZDV group. This was accompanied by activation of the PINK1/PTEN-dependent mitophagy pathway, prevention of cf-mtDNA release, and restoration of gut microbial diversity. In contrast, the *Lactobacilli* mixture not only failed to improve outcomes but was associated with exacerbated intestinal damage, more pronounced cognitive dysfunction, and no reduction in neuroinflammatory markers. **Conclusions:** Combined exposure to LPS and ZDV induces gut–brain axis dysfunction characterized by neuroinflammation, cognitive impairment, intestinal damage, and dysbiosis. *Bacillus subtilis* effectively preserves cognitive function through activation of PINK1/PTEN-dependent mitophagy and suppression of neuroinflammation, highlighting its potential as a therapeutic candidate for cognitive impairments associated with gut–brain axis dysfunction. The contrasting effects of the lactobacilli mixture underscore the critical importance of strain-specificity in probiotic interventions.

## 1. Introduction

Systemic chronic inflammation is the primary cause of chronic degenerative diseases, including diabetes, cancer, cardiovascular, autoimmune diseases, and neurodegenerative ones [1]. Chronic inflammation is considered a significant factor contributing to neurodegeneration and progressive cognitive decline [2]. There are several methods for modeling both acute and chronic inflammation in rodents. Transgenic models are distinguished, for example, human leukocyte antigen (HLA)-transgenic mouse models, where stable activation of inflammatory processes was observed, chemical models, for example, the use of carrageenan, immunological models, for example, complete Freund’s adjuvant [3], and bacterial models, in particular, lipopolysaccharide (LPS). LPS is an endotoxin of the outer membrane of Gram-negative bacteria and is a potent proinflammatory agent. Its biological action is mediated by binding to Toll-like receptor 4 (TLR4), which triggers a cascade reaction and the secretion of key proinflammatory cytokines. Due to its ability to reliably induce a systemic inflammatory response, LPS is widely used in experimental models to study pathologies, including those of the respiratory system [4].

There are contradictory data regarding the effect of LPS in various experimental models. Despite the fact that a significant portion of studies showed memory impairment after LPS administration to rodents [5,6,7,8,9,10], there are data indicating the opposite. Intraperitoneal injections of LPS to mice at a concentration of 375 μg/kg did not lead to an increase in platform search time in the Morris water maze when assessing long-term spatial memory [11,12]. It was shown that subcutaneous administration of LPS (50 μg/kg) on the 3rd and 5th days of life contributed to selective impairment of long-term memory in males, but not in females, and also had no effect on short-term spatial memory [13]. A study on rats demonstrated that a single neonatal administration of LPS (P5, P30) selectively impaired long-term spatial memory in the probe trial and contextual fear memory, without affecting the learning process [14]. Acute LPS-stress (a single administration of 350 μg/kg) improved spatial learning in the Morris water maze, and rats spent less time in the peripheral zone of the pool, indicating less anxiety. Chronic LPS-stress (7-day administration of 200 μg/kg) impaired only short-term memory, but no statistically significant differences were revealed, and it did not affect long-term memory in any way [13]. Thus, at present, there is no consensus on the effect of LPS-induced inflammation on memory in experimental rodent models.

Disturbances of the gut microbiome and the subsequent rise in bacterial endotoxin levels, including LPS, are regarded as a critical pathogenic link negatively impacting cognitive functions through the induction of systemic and neuroinflammation [15]. It is noteworthy that LPS frequently serves as a concomitant factor capable of exacerbating the toxic effects of other compounds. Previous studies have demonstrated that LPS exacerbates functional and inflammatory responses to ovalbumin [16] and intensifies oxidative stress during thrombin administration [17].

In the present study, zidovudine (ZDV)—a compound with distinct antibacterial activity—is employed to specifically modulate the gut–brain axis and induce its dysfunction. Despite ZDV being a classic nucleoside reverse transcriptase inhibitor used in antiviral therapy, it, like other antibacterial agents, exerts a significant influence on the composition and function of the intestinal microbiota [18]. Importantly, the combination of LPS and ZDV is highly relevant to the clinical context of HIV infection, where patients often exhibit both elevated systemic LPS levels due to microbial translocation from a damaged gut and undergo long-term ZDV treatment. It has been shown that plasma levels of bacterial 16S rDNA (a marker of microbial translocation) remain elevated in HIV-infected individuals despite antiretroviral therapy, and these levels correlate with immune activation and impaired CD4+ T-cell restoration [19]. Furthermore, the combined use of LPS and ZDV is not without precedent, as previous in vitro studies have specifically investigated the effects of zidovudine on LPS-stimulated immune cells [20]. Experimental strategies for investigating the gut–brain axis frequently include the manipulation of the microbiota via antiretroviral therapy drugs [21], enabling the modeling of dysbiosis and the study of its impact on the central nervous system.

Thus, given that the combined administration of LPS and ZDV targets the induction of systemic inflammation and the disruption of the gut microbiome—key components of the gut–brain axis—attempting to modulate these cognitive impairments through the use of probiotics is a logical progression. In this study, two distinct approaches were employed for correction. The first is based on the application of the spore-forming bacterium *Bacillus subtilis*. It has been previously demonstrated that *B. subtilis* can mitigate LPS-induced immune stress and oxidative imbalance, as well as counteract mitochondrial damage [22]. The second approach involves the use of a combination of lactobacilli (*Lactobacillus helveticus*, *L. plantarum*, and *L. paracasei*). These strains were selected based on scientific data indicating their positive impact on cognitive functions [23]. Furthermore, these strains are associated with resistance to gastrointestinal infections, enhanced pathogen defense, immune response modulation [24], improved beta-diversity [25], normalization of glucose metabolism, and enhanced host cell ATP availability [26].

The aim of this study was a comprehensive assessment of the protective potential of two probiotic preparations regarding gut–brain axis disorders induced by the combined administration of LPS and ZDV. To this end, the objectives were to evaluate the influence of the combined exposure to LPS and ZDV on the spatial memory of mice in the Morris water maze test, study the changes in the gut microbiome composition and the histological structure of the small intestine induced by this exposure, and compare the efficiency of the correction of the identified disorders using the probiotics *B. subtilis* and a combination of *L. helveticus*, *L. plantarum*, *L. paracasei*.

## 2. Materials and Methods

### 2.1. Animals: Experimental Design

Male C57BL/6 mice aged 2 months, which were obtained from the “Stolbovaya” nursery (Moscow region, Russia), were used in the experiment. All experiments conducted with laboratory animals were reviewed and approved by the Ethical Committee for Biomedical Research of Voronezh State University (Section of Animal Care and Use, protocol No. 5001-01 dated 11 December 2025). The mice were kept under standard conditions: t = 25 °C, a 12 h light day and relative humidity of at least 40%, no more than 6 mice per cage.

Mice were randomly assigned to 5 groups in a blinded manner. The 1st group (Control, *n* = 12) was subjected to injections of physiological saline from the 9th to the 15th day of the experiment and received standard food and water throughout the entire experiment. Mice from the 2nd group (LPS group, *n* = 12, at the end of the experiment *n* = 11) received standard food and water throughout the entire experiment, and they were also administered intraperitoneal injections of LPS (commercial preparation “Pyrogenal” The Gamaleya national center of epidemiology and microbiology, Russia) at a dose of 375 μg/kg/day from the 9th to the 15th day of the experiment. The 3rd group of mice (LPS + ZDV group, *n* = 12) was subjected to intraperitoneal injections of LPS at a dose of 375 μg/kg/day, and every 24 h the mice orally received the drug “Zidovudine” (LLC “Atoll”, Russia) at a dose of 10 mg/mL, as well as standard food during the entire experiment. The 4th group of mice (LPS + ZDV + *B. subtilis*, *n* = 12) was subjected to intraperitoneal injections of LPS at a dose of 375 μg/kg/day, and every 24 h the mice orally received ZDV at a dose of 10 mg/mL and food mixed with the probiotic *Bacillus subtilis* at a rate of 19 g of probiotic per 180 g of food during the entire experiment. Mice from the 5th group (LPS + ZDV + *Lactobacillus* spp., *n* = 12) were subjected to intraperitoneal injections of LPS at a dose of 375 μg/kg/day, and every 24 h the mice orally received ZDV at a dose of 10 mg/mL and food mixed with a probiotic consisting of a combination of *Lactobacillus helveticus*, *Lactiplantibacillus plantarum* (until 2020 known as *Lactobacillus plantarum*, *Lacticaseibacillus paracasei* (until 1989 known as *Lactobacillus paracasei*) at a rate of 19 g of probiotic (10^9^ CFU/g of each species bacteria) per 180 g of food during the entire experiment. The daily food consumption by mice was 4.9 ± 0.09 g (each mouse consuming on average 4.9 × 10^8^ bacteria daily). This dose of bacteria is generally accepted when studying the effects of probiotic bacteria on animals [27,28,29].

**Figure 1:**
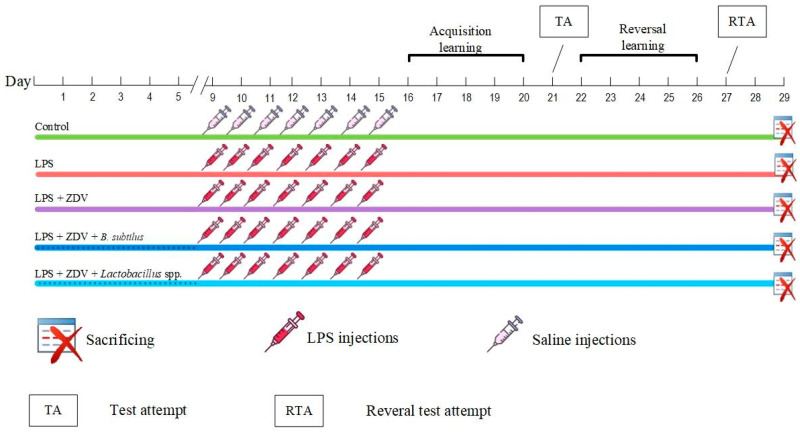
Experimental design. Control, *n* = 12; LPS, *n* = 12; LPS + ZDV, *n* = 12; LPS + ZDV + *B. subtilis*, *n* = 12; LPS + ZDV + *Lactobacillus* spp., *n* = 12.

The Morris water maze began on the 16th day of the experiment and continued for 12 days. Twenty-eight days after the start of the experiment, the mice were sacrificed. Before sacrifice, the gut microbiome was collected and immediately frozen. The mice were anesthetized via intraperitoneal injection of a mixture of Zoletil 50 (a combination of tiletamine and for zolazepam) (Virbac, Carros, France) at a concentration of 25 mg/kg and Xylazine (NITA-FARM, Saratov, Russia) at a concentration of 7.5 mg/kg [30]. After anesthetization and prior to sacrifice, blood was collected from the mice by puncturing the retro-orbital eye sinus. A portion of the whole blood was used for the preparation of cytological specimens. The remaining blood was immediately separated into plasma and formed elements. After a 15 min incubation at room temperature, the whole blood was centrifuged for 5 min at 3500× *g*. Subsequently, the mice were sacrificed by cervical dislocation followed by decapitation. After decapitation, the brain was removed and immediately frozen. The liver and intestines were also removed. The liver was weighed, and the length of the intestine was measured. A portion of the small intestine was placed in formalin for storage and subsequent preparation of histological specimens (Figure 1).

### 2.2. Morris Water Maze

The experimental apparatus for studying spatial long-term memory in mice was the Morris water maze: a circular pool measuring 154 cm in diameter, with a 15 cm circular platform located in the target quadrant. To render the platform invisible, the pool was filled with water made opaque using white coloring, with the platform positioned 0.5 cm beneath the water’s surface. The mouse launch protocol was conducted according to the described protocol by Vorhees (2006) [31]. The training trial was conducted for 5 days, with 4 trials per day, each lasting a maximum of 1 min. If a mouse failed to locate the platform, it was manually placed onto it and allowed to remain there for 15 s. On the 6th day, a trial was conducted to assess spatial long-term memory. One trial lasting one minute was given to search for the platform. In the next 5 days, reverse learning took place, and the platform was positioned in the quadrant opposite to the original one. On the 12th day, a reverse trial was also conducted. Starting points corresponded to the protocol [31] presented in Table 1. Two parameters were evaluated. The first was the total time (in seconds) that the mouse spent searching for the platform during the second trial. If the mouse failed to locate the platform within this trial, a maximum time of 60 s was recorded. The second parameter was the distance swam by the while searching for the platform (in millimeters). If the platform was not found within the allotted time, the distance length for the trial was not indicated.

### 2.3. Measurement of the Number of Copies and the Amount of mtDNA Damage

DNA from mitochondria was obtained using the “Proba-GS” DNA extraction kit (“DNA-Technology”, Moscow, Russia). The relative mitochondrial DNA (mtDNA) copy number was assessed using real-time Polymerase Chain Reaction (PCR) on the Real-Time CFX96™ platform (Bio-Rad, Hercules, CA, USA) in the cortex and plasma. Primers used for the amplification of mtDNA (mitoShort) and a nuclear DNA region (NuclShort), relative to which normalization was performed. Reaction conditions: total denaturation at 95 °C for 3 min, then 35 cycles: denaturation at 95 °C for 10 s, primer annealing at 59 °C for 30 s, elongation at 72 °C for 30 s. Calculation of the relative mtDNA copy number was performed using the standard Bio-Rad CFX Manager software (version 2.1) based on the standard formula 2^(−ΔΔCq)^.

The level of mtDNA damage was assessed using long-range PCR. Amplification was performed with the En-cycle polymerase kit (Evrogen, Moscow, Russia) on a CFX96™ Real-Time System thermal cycler (Bio-Rad, USA). The underlying principle is that DNA lesions impede polymerase processivity, resulting in a reduced rate of product accumulation. The PCR cycling conditions were as follows: initial denaturation at 95 °C for 3 min; followed by 35 cycles of denaturation at 95 °C for 30 s, annealing at 59 °C for 30 s, and elongation at 72 °C for 4 min 30 s. To quantify damage, the difference in quantification cycle values (ΔCq) between control and experimental samples for the long fragment was compared to the ΔCq for the short fragment. The sequences of the primers are presented in Table 2. The amount of additional mtDNA damage was calculated per 10 kb using the following formula:

(1) Lesions = (1 − 2^(−(∆long − ∆short))) × ((10,000 (bp))/(fragment length (bp))

where Δlong = Cq control − Cq experiment for the long fragment and Δshort = Cq control − Cq experiment for the short fragment.

### 2.4. Gene Expression Assessment

Total RNA was extracted from brain tissue using the ExtractRNA kit (Evrogen, Russia) in accordance with the manufacturer’s instructions. Reverse transcription was carried out on an Eppendorf Mastercycler personal thermal cycler (Eppendorf, Enfield, CT, USA) employing the REVERTA-L kit (AmpliSens, Moscow, Russia) following the supplied protocol. Quantitative PCR (qPCR) was subsequently performed on a CFX96™ Real-Time thermal cycler (Bio-Rad, USA) using the qPCRmix-HS SYBR kit (Evrogen, Russia). The normalized expression level of target genes was calculated with Bio-Rad CFX Manager software (version 2.1) applying the standard 2^−ΔΔCq^ method. The *Gapdh* gene was used as a reference. Primer sequences of the studied genes are presented in Table 3.

### 2.5. Assessment of the Bacterial Composition of the Intestinal Microbiome

Total DNA was extracted from each fecal sample using the K-SORB-50 spin column kit (Syntol, Moscow, Russia) according to the manufacturer’s protocol for biological material.

Afterwards, each DNA sample was fragmented with subsequent ligation of adapters. These manipulations were performed using commercial kits MGIEasy Fast FS Library Prep Module (MGI, Shenzhen, China) and MGIEasy UDB Adapter Kit (MGI, China) in accordance with the manufacturer’s protocols. Further, quality control of the obtained libraries was carried out using the method of electrophoresis in a 2% agarose gel. Also, the concentration of the sequencing libraries was measured using a Qubit 2.0 r fluorometer (Thermo Fisher, Waltham, MA, USA) and the QuDye^®^ HS kit for determining the amount of double-stranded DNA (Lumiprobe, Moscow, Russia). Based on the results obtained, the libraries were combined into pools.

Then, the obtained pools were circularized according to the manufacturer’s protocol of the commercial kit MGIEasy Dual Barcode Circularization Module (MGI, China). At this stage, quality control was also carried out by assessing the concentration using a Qubit 2.0 fluorometer (Thermo Fisher, USA) and the QuDye^®^ HS kit for determining the amount of double-stranded DNA (Lumiprobe, Russia). Based on the quality control results, the volume of each pool for combining into a superpool was calculated. The concentration of the superpool was also monitored, and its volume was calculated as 60 fmol.

To start the DNBSEQ-G50 sequencer (MGI, China), DNA-nanoballs (DNBs) were formed using a commercial high-throughput sequencing kit DNBSEQ-G50RS FCL PE100/FCS PE150 (MGI, China). Then, the concentration of the obtained DNBs was measured using a Qubit 2.0 fluorometer (Thermo Fisher, USA) and a commercial single-stranded DNA analysis kit QuDye (Lumiprobe, Russia). After this, the DNBs were placed into the sequencer using a DNBSEQ-G50RS Sequencing Flow Cell FCL (MGI, China), as well as a reagent cartridge from the DNBSEQ-G50RS High-throughput Sequencing Kit FCL PE100/FCS PE150 (MGI, China). All operations were performed according to the manufacturer’s protocols.

The quality of the raw metagenomic data was evaluated using FastQC (v0.12.1). Adapter and other technical sequences were removed with flexbar (v3.5.0). Reads originating from the host were depleted by aligning the metagenomic sequences against the human (GCF000001405.40) and mouse (GCF000001635.27) reference genomes using Bowtie2. Subsequently, taxonomic profiling of the samples was performed with MetaPhlAn 4 (v4.1.1), utilizing its default databases for bacterial, viral, and eukaryotic markers.

### 2.6. Histological Studies of the Intestine

For morphological examination of the small intestine, tissue specimens were fixed in a 10–12% neutral formalin solution (HistoSafe^®^, Biovitrum, B06-003/5, Moscow, Russia). Following fixation, the intestinal tissue was dehydrated through a graded ethanol series and embedded in Histomix histological paraffin (Biovitrum, 247, Moscow, Russia). Sections of 3–5 μm in thickness were cut from the paraffin blocks using an MPS-2 rotary microtome (Tochmedpribor, 00460, Kharkiv, Ukraine). The obtained sections were subsequently stained using conventional histological techniques, namely hematoxylin and eosin. The sections were deparaffinized by incubation in two successive baths of O-xylene (Ekos-1, 1330-20-7, Moscow, Russia) for 15 min each, and subsequently rehydrated in two changes of 96% alcohol. Then, the sections were stained with Mayer’s hematoxylin (Biovitrum, 05-001, Moscow, Russia) for 15 min. The stained sections were then washed under running water for 20 min. The staining procedure began with a brief rinse in running water, after which the sections were immersed in a wide-mouth flask containing 1% aqueous-alcoholic eosin (Labico, Labico LLC, E-013/1000, Saint Petersburg, Russia) for 5 s. Excess stain was removed by washing in distilled water for 30 s. Next, the sections were dehydrated in 96% alcohol for 10 min and subsequently cleared in O-xylene for 10 min. “Mounting” of the stained histological sections was performed using HISTOPOINT mounting medium (MedTechnikaPoint LLC, 191119, Saint Petersburg, Russia). Evaluation of tissue architecture was carried out using light microscopy on a Biomed 4 microscope (Biomed, 00000023514, Moscow, Russia).

### 2.7. Evaluation of the Blood Leukocyte Formula

For morphological study of peripheral blood, fresh whole blood samples stabilized with an anticoagulant (sodium citrate) were used. Smears were prepared using the “spatula” method: 3.7 μL of blood was applied to a degreased slide, and a second slide was used to distribute the drop at an angle of 30–35° until a thin monolayer of cells was formed in the distal part of the smear (“feather”). The resulting preparations were air-dried, fixed with 95% methyl alcohol for 5 min, and stained with May–Grünwald combined Eosin Methylene Blue stain (MiniMed^®,^ Bryansk, Russia). After washing with running water, the smears were air-dried.

Morphological evaluation and leukocyte counting were performed using a LOMO Micmed-6 light microscope (JSC “LOMO”, Saint Petersburg, Russia). The optimal zone for analysis was selected at low magnification, and the differential cell count was performed at 40× magnification in the thin part of the smear, considering only whole cells. The leukocyte formula was determined by counting ≥ 100 cells with differentiation into segmented and band neutrophils, lymphocytes, monocytes, eosinophils, and basophils. Morphological features of cells, including signs of immaturity and toxic granulation, were also recorded.

### 2.8. Statistical Analysis

The results obtained during the experiment are presented as mean values ± standard error. Statistical processing of the data was performed using the GraphPad Prism 8.4.3 software package. To assess the reliability of differences between groups, the non-parametric Kruskal–Wallis analysis of variance with Dunn’s post hoc test was used. For repeated measurements within the same group over time (body weight dynamics), the Wilcoxon matched-pairs signed-rank test was applied. The paper discusses statistically reliable differences (*p* < 0.05).

Correlation analysis was performed in the Jupyter Notebook v7 environment based on the calculation of the programmable Spearman rank correlation. Results were considered statistically significant at a *p* value ≤ 0.01 with adjustment for multiple comparisons performed using the Bonferroni correction.

Statistical analysis of sequencing data was performed in the R environment (Version 4.4.1). Alpha-diversity was assessed using the Shannon index. To identify differences in alpha-diversity, the non-parametric Mann–Whitney test was applied. Beta-diversity analysis was conducted based on the Bray–Curtis dissimilarity metric. To estimate differences in diversity between groups, we used the ADONIS function (999 permutations). To ensure that the observed effects reflect genuine location shifts rather than uneven distribution within groups, we supplemented ADONIS with the PERMDISP function (999 permutations). Differences in the relative abundance of bacterial species between groups were detected using the ANCOMBC method (version 2.6.0) with the FDR (Benjamini-Hochberg) method and a significance threshold of α = 0.05. Results were considered statistically significant at an adjusted *p*-value ≤ 0.05. Sequencing results are presented as mean values ± standard deviation (SD).

## 3. Results

### 3.1. Effect of ZDV, LPS, and Probiotics on Morphological Parameters of Mice

**Figure 2:**
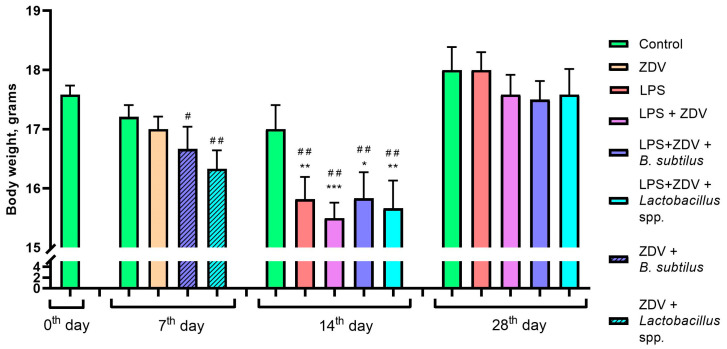
Effect of ZDV, LPS, and probiotics on body weight dynamics throughout the experiment. Control, *n* = 12; LPS, *n* = 11; LPS + ZDV, *n* = 12; LPS + ZDV + *B. subtilis*, *n* = 12; LPS + ZDV + *Lactobacillus* spp., *n* = 12. Differences from the control are statistically significant: * *p* < 0.05, ** *p* < 0.01, *** *p* < 0.001 (Kruskal–Wallis test, Dunn’s post hoc test). Differences within experimental groups compared to day 0 are statistically significant: ^#^ *p* < 0.05, ^##^ *p* < 0.01 (Wilcoxon matched-pairs test).

Preliminary administration of ZDV for 7 days did not have a statistically significant effect on changes in mouse body weight during therapy. However, in the subsequent week, LPS injections led to a significant decrease in body weight (at least, *p* < 0.05). The body weight of mice in the experimental groups receiving LPS injections together with ZDV therapy decreased on average from 6.9% to 8.8%. By 28 days after the start of the experiment, the body weight of the mice normalized, which may indicate adaptation of the organism to the toxicants (Figure 2). The Wilcoxon matched-pairs test showed that on day 7, the differences within the control group and the group of mice receiving ZDV alone were not statistically significant compared to the pre-treatment period. However, in mice that received *B. subtilis* or *Lactobacillus* spp. in addition to ZDV, body weight was significantly reduced (*p* < 0.05 and *p* < 0.01, respectively). On day 14, differences in body weight within the control group were not statistically significant compared to the pre-experimental period (*p* = 0.67). LPS alone, as well as the LPS + ZDV combination, induced a statistically significant decrease in body weight by day 14 (both *p* < 0.01). Similarly, significant differences were found in the groups that additionally received LPS + ZDV + *Lactobacillus* spp. (*p* < 0.01) and LPS + ZDV + *B. subtilis* (*p* < 0.01) (Figure 2).

**Figure 3:**
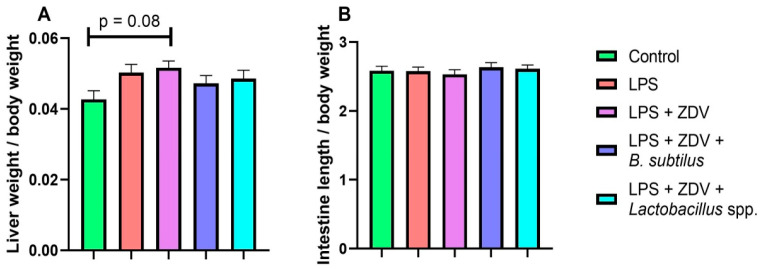
Effect of ZDV, LPS, and probiotics on liver mass (**A**) and intestine length (**B**). Control, *n* = 12; LPS, *n* = 11; LPS + ZDV, *n* = 12; LPS + ZDV + *B. subtilis*, *n* = 12; LPS + ZDV + *Lactobacillus* spp., *n* = 12.

The LPS + ZDV combination was sufficiently toxic for the mice, and a tendency toward an increase in liver weight by 20% in the LPS + ZDV experimental group compared to the control was observed; however, the values were not statistically significant (*p* = 0.08) (Figure 3A). No significant differences in intestine length relative to body weight were found (Figure 3B).

### 3.2. Effect of ZDV, LPS, and Probiotics on Spatial Memory

**Figure 4:**
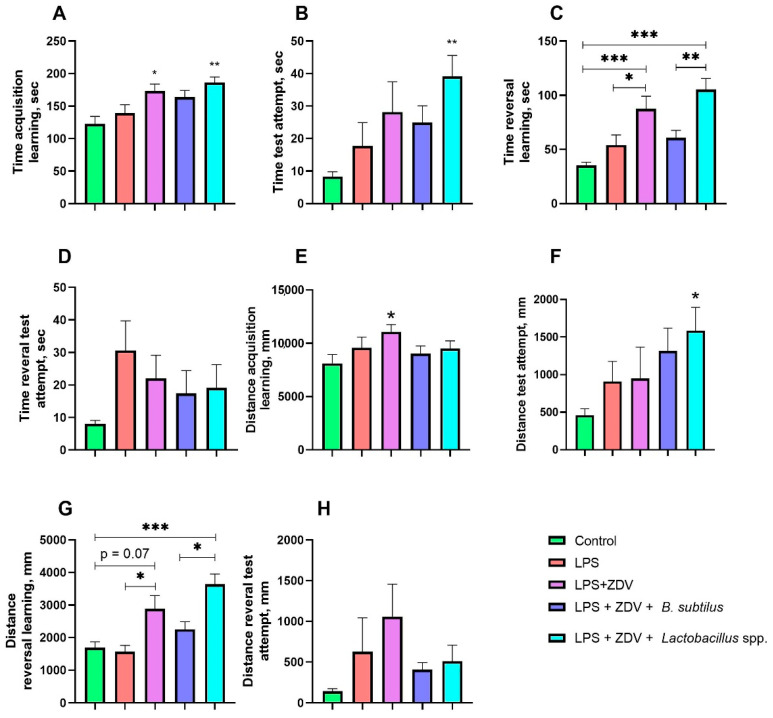
Time spent by mice searching for the platform during the Morris test: during forward learning (**A**), on experimental day 6 (**B**), during reverse learning (**C**), on experimental day 12 (**D**). Distance traveled by mice during the Morris test: during forward learning (**E**), on experimental day 6 (**F**), during reverse learning (**G**), on experimental day 12 (**H**). Control, *n* = 12; LPS, *n* = 11; LPS + ZDV, *n* = 12; LPS + ZDV + *B. subtilis*, *n* = 12; LPS + ZDV + *Lactobacillus* spp., *n* = 12. Differences between experimental groups are statistically significant: * *p* < 0.05, ** *p* < 0.01, *** *p* < 0.001 (Kruskal–Wallis test, Dunn’s post hoc test).

We analyzed the time mice spent searching for the platform during test days and training days. We analyzed the time mice spent searching for platforms in four attempts during training days (from day 1 to day 5). A significant increase in the time to find the platform was detected in the LPS + ZDV groups by 41.3% (*p* < 0.05) and LPS + ZDV + *Lactobacillus* spp. by 51.8% (*p* < 0.01) compared to the control (Figure 4A). In the test attempt, on the 6th day of performing Morris Water Maze (MWM), we found a tendency towards an increase in the time spent searching for the platform in the LPS + ZDV experimental groups by approximately 3 times on the 6th experimental day; however, significant differences were observed only when comparing the control group with LPS + ZDV + *Lactobacillus* spp. (*p* < 0.01) (Figure 4B).

Similarly, during reversal learning, a more than two-fold increase in the time spent searching for the platform was observed (*p* < 0.001). It is worth noting that mice in the experimental group that received *B. subtilis* in addition to LPS + ZDV showed a decrease in time by approximately 30% compared to the control group (Figure 4C). No differences in the time spent searching for the platform on experimental day 12 were found (Figure 4D).

Similar results were obtained when analyzing the distance mice swam in search of the platform. During forward learning, mice from the LPS + ZDV experimental group covered a distance 26.7% greater than mice from the control group (*p* < 0.05); however, probiotic consumption negated this increase in distance (Figure 4E). Nevertheless, on the 6th test day, the greatest distance was covered by mice from the LPS + ZDV + *Lactobacillus* spp. group (71% greater compared to the control group (*p* < 0.05)) (Figure 4F). During reverse learning, the greatest distance was swum by mice from the LPS + ZDV and LPS + ZDV + *Lactobacillus* spp. experimental groups, by 41.3% (*p* = 0.07) and 53.4% (*p* < 0.001), respectively, compared to the control, while mice receiving *B. subtilis* swam a distance 38% shorter (*p* < 0.05) compared to the LPS + ZDV + *Lactobacillus* spp. experimental group (Figure 4G). On the 12th test day, no significant differences between groups were found (Figure 4H).

### 3.3. Effect of ZDV, LPS, and Probiotics on Mitochondrial Functions and Inflammatory Processes

**Figure 5:**
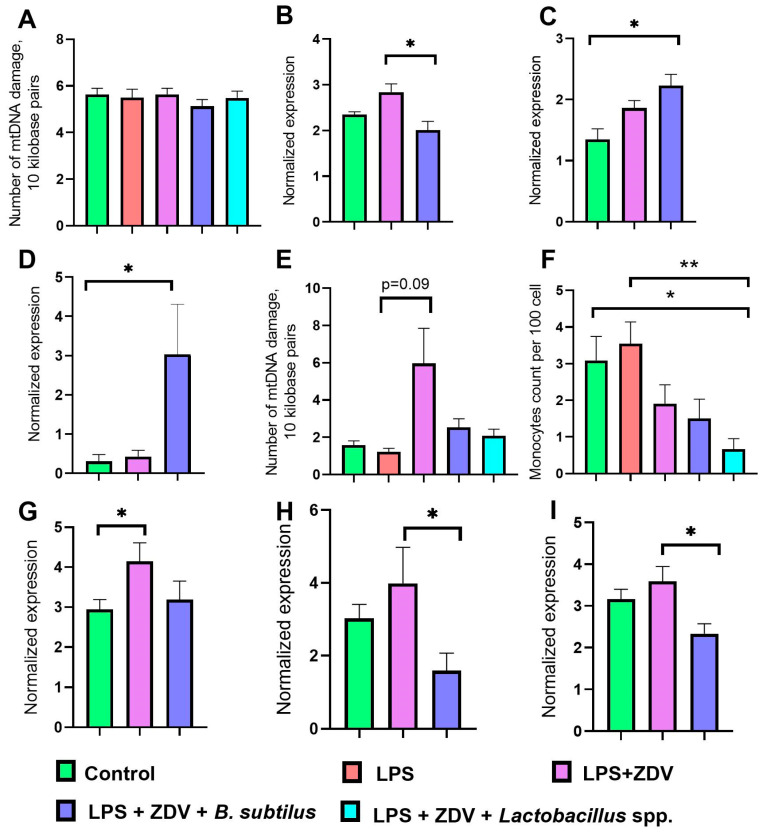
Effect of LPS, ZDV, and probiotic mixtures on the amount of mtDNA damage (**A**), level of *Fis1* gene expression (**B**), *Pink1* (**C**), *Pten* (**D**) in the cerebral cortex, as well as the level of cf-mtDNA (**E**) and the number of monocytes in mouse blood (**F**), level of Thfagene expression (**G**), *Il1b* (**H**), *Il6* (**I**) in the cerebral cortex. For (**A**,**E**,**F**): Control, *n* = 12; LPS, *n* = 11; LPS + ZDV, *n* = 12; LPS + ZDV + *B. subtilis*, *n* = 12; LPS + ZDV + *Lactobacillus* spp., *n* = 12. For (**B**–**D**,**G**–**I**): Control, *n* = 5; LPS + ZDV, *n* = 5; LPS + ZDV + *B. subtilis*, *n* = 5. Differences between experimental groups are statistically significant: * *p* < 0.05, ** *p* < 0.01 (Kruskal–Wallis test, Dunn’s post hoc test).

Although ZDV is considered a strong damaging factor for mtDNA, we did not detect changes in the amount of mtDNA damage (Figure 5A). At the same time, mice receiving combined LPS + ZDV therapy showed a tendency towards increased expression of the Fis gene, responsible for mitochondrial fission. However, in mice that, in addition to combined therapy, received the probiotic mixture *B. subtilis*, the level of mitochondrial fission 1 protein (*Fis 1*) gene expression was 30% lower (*p* < 0.05) (Figure 5B). Furthermore, administration of *B. subtilis* contributed to an almost two-fold increase in the expression of the PTEN Induced Kinase 1 (*Pink1*) gene relative to the control (*p* < 0.05) (Figure 5C). Alongside this, administration of *B. subtilis* contributed to a 9-fold increase in the expression of the phosphatase and TENsin homolog (*Pten*) gene (*p* < 0.05) compared to the control group (Figure 5D).

The concentration of cell-free mitochondrial DNA (cf-mtDNA) in the blood plasma of mice after combined LPS + ZDV therapy increased 5-fold compared to mice receiving LPS monotherapy; however, the differences were not statistically significant (*p* = 0.09). Meanwhile, in mice that, in addition to combined therapy, received the probiotic mixture, the level of cf-mtDNA was almost at the level of control values (Figure 5E). For example, the concentration of monocytes decreased 5-fold in the LPS + ZDV + *Lactobacillus* spp. group compared to the group of mice receiving LPS monotherapy (Figure 5F).

The combination of LPS + ZDV led to a significant increase in the expression of tumor necrosis factor-alpha (*Tnfa*) in the brain (*p* < 0.05), whereas in the group of mice that additionally received the *B. subtilis* probiotic mixture, *Tnfa* expression levels remained at control levels (Figure 5G). In the group of mice treated with the LPS + ZDV combination, there was also a trend toward increased expression of interleukin-1 beta (*Il1b*) and interleukin-6 (*Il6*); however, the differences from the control were not statistically significant. In mice receiving *B. subtilis*, the expression of both genes was reduced compared to the LPS + ZDV group (both *p* < 0.05) (Figure 5H,I).

### 3.4. Effect of ZDV, LPS, and Probiotics on the Bacterial Composition of the Gut Microbiome

Analysis of the fecal microbiome of laboratory mice from five experimental groups allowed for the identification of 10 phyla, 51 classes, 55 orders, 69 families, 227 genera, and 320 species of bacteria.

**Figure 6:**
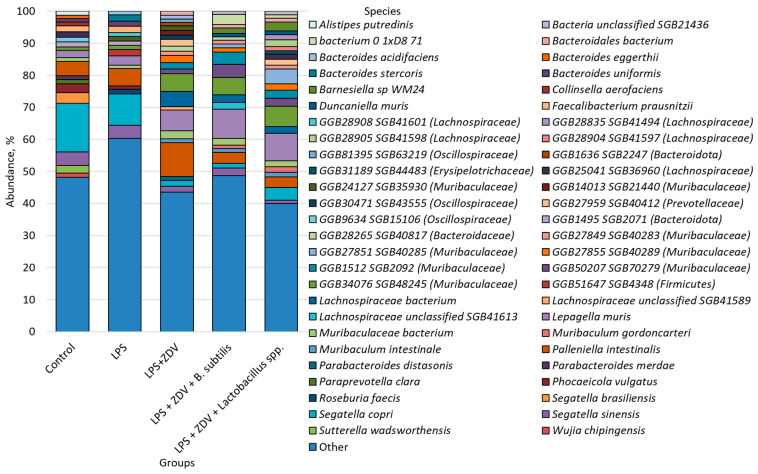
Bacterial species detected in the microbiome of experimental groups. Control, *n* = 5; LPS, *n* = 6; LPS + ZDV, *n* = 6; LPS + ZDV + *B. subtilis*, *n* = 6; LPS + ZDV + *Lactobacillus* spp., *n* = 6.

We conducted a comparative analysis of the microbiome of the studied groups, during which we identified 52 of the most common species, the abundance of which exceeded 1%; all other species were grouped as “Other” (Figure 6).

**Figure 7:**
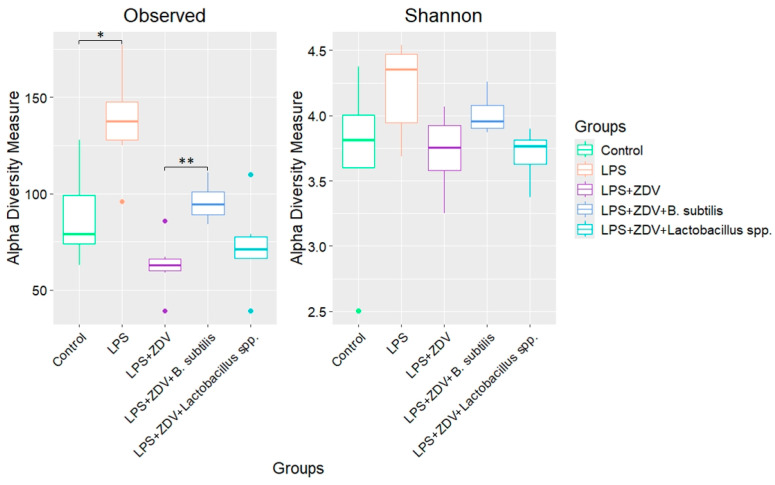
Alpha-diversity of the microbiome of the studied groups. Control, *n* = 5; LPS, *n* = 6; LPS + ZDV, *n* = 6; LPS + ZDV + *B. subtilis*, *n* = 6; LPS + ZDV + *Lactobacillus* spp., *n* = 6. Differences between experimental groups are statistically significant: * *p* < 0.05, ** *p* < 0.01 (Bonferroni test).

An analysis of alpha-diversity was conducted using the observed species diversity measure and the Shannon index. Statistically significant differences were identified only in the number of observed species between the control group and mice receiving LPS (88.6 ± 25.6 vs. 137.3 ± 26.9, *p* < 0.05). It was also found that administration of *B. subtilis* significantly increased the number of species relative to the LPS + ZDV group (95.7 ± 9.9 vs. 62.8 ± 15.1, *p* < 0.01) (Figure 7).

**Figure 8:**
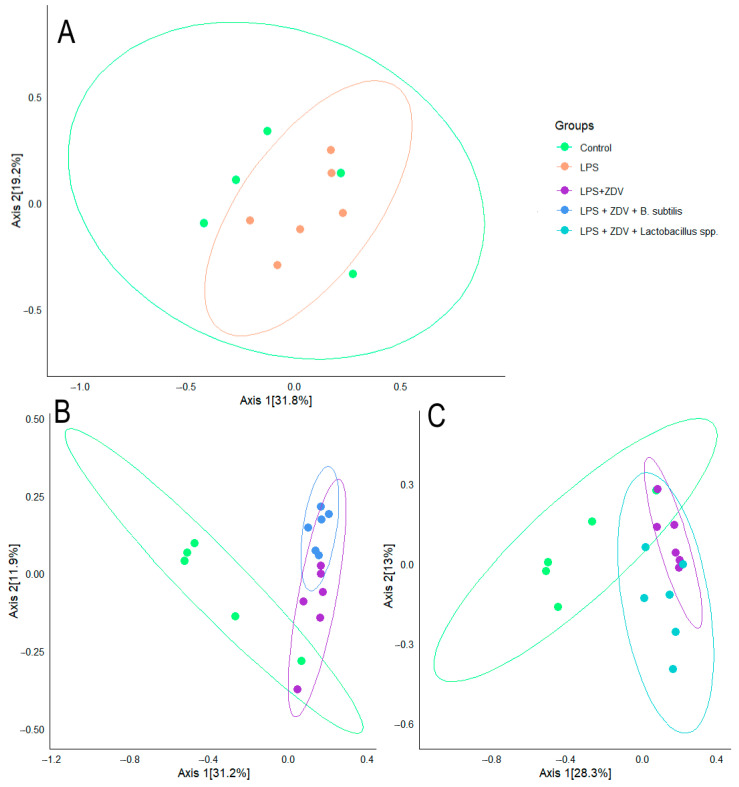
Principal Coordinates Analysis (PCoA) plot of beta-diversity based on the Bray–Curtis scale between experimental groups: Control and LPS (**A**); Control, LPS + ZDV, LPS + ZDV + *B. subtilis* (**B**); Control, LPS + ZDV, LPS + ZDV + *Lactobacillus* spp. (**C**). Control, *n* = 5; LPS, *n* = 6; LPS + ZDV, *n* = 6; LPS + ZDV + *B. subtilis*, *n* = 6; LPS + ZDV + *Lactobacillus* spp., *n* = 6.

Analysis of beta-diversity also showed the presence of clustering between the studied groups. The centroid of the microbiome of the control group and the group of mice receiving LPS injections coincided (Figure 8A). Meanwhile, the centroid of the microbiome of the control group was statistically significantly different from the LPS + ZDV group (*p* = 0.003), from the LPS + ZDV + *B. subtilis* group (*p* = 0.003), and from the LPS + ZDV + *Lactobacillus* spp. group (*p* = 0.003) (Figure 8B,C).

**Figure 9:**
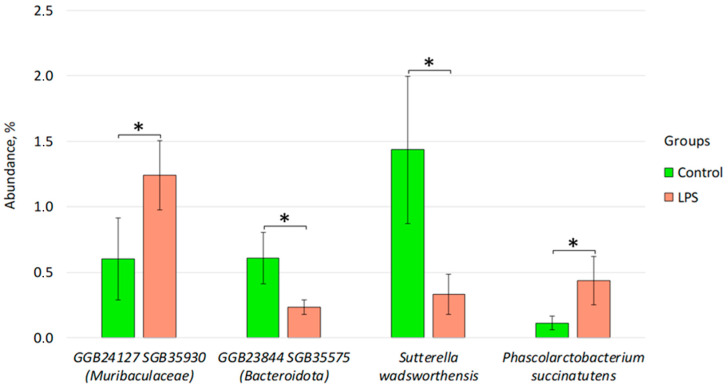
Differences in the composition of the fecal microbiome between the control group and mice receiving LPS injections. Control, *n* = 5; LPS, *n* = 6; LPS + ZDV, *n* = 6; LPS + ZDV + *B. subtilis*, *n* = 6; LPS + ZDV + *Lactobacillus* spp., *n* = 6. Differences between experimental groups are statistically significant: * *p* < 0.05 (Bonferroni test).

Differential abundance analysis revealed statistically significant differences at the species level between 13 of the 320 species tested. In the LPS group compared to the control group, a decrease in the abundance of species was observed: GGB23844 SGB35575 (phyla *Bacteroidota*) (from 0.61% ± 0.19 to 0.23% ± 0.05, *p* < 0.05), *Sutterella wadsworthensis* (from 1.43% ± 0.56 to 0.33% ± 0.15, *p* < 0.05) and an increase in the abundance of species: GGB24127 SGB35930 (family *Muribaculaceae*) (from 0.60% ± 0.31 to 1.24% ± 0.26, *p* < 0.05), *Phascolarctobacterium succinatutens* (from 0.11% ± 0.05 to 0.44% ± 0.18, *p* < 0.05) (Figure 9).

**Figure 10:**
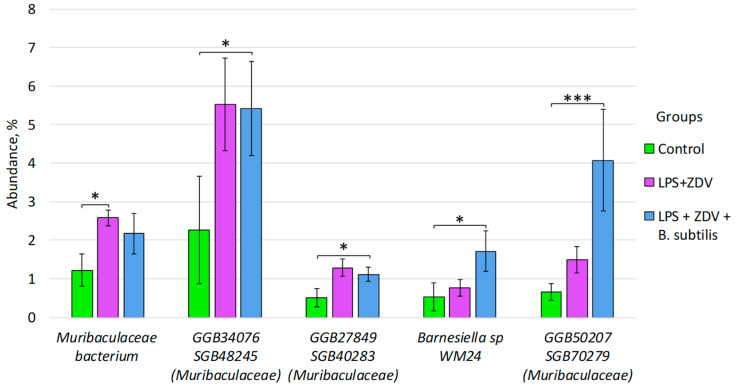
Differences in the composition of the fecal microbiome between the Control, LPS + ZDV, LPS + ZDV + *B. subtilis* groups. Control, *n* = 5; LPS, *n* = 6; LPS + ZDV, *n* = 6; LPS + ZDV + *B. subtilis*, *n* = 6; LPS + ZDV + *Lactobacillus* spp., *n* = 6. Differences between experimental groups are statistically significant: * *p* < 0.05, *** *p* < 0.001 (Bonferroni test).

**Figure 11:**
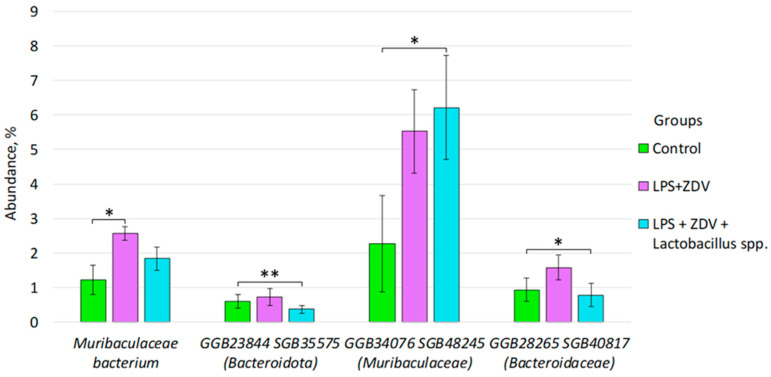
Differences in the composition of the fecal microbiome between the Control, LPS + ZDV, and LPS + ZDV + *Lactobacillus* spp. groups. Control, *n* = 5; LPS, *n* = 6; LPS + ZDV, *n* = 6; LPS + ZDV + *B. subtilis*, *n* = 6; LPS + ZDV + *Lactobacillus* spp., *n* = 6. Differences between experimental groups are statistically significant: * *p* < 0.05, ** *p* < 0.01 (Bonferroni test).

Statistically significant differences were also identified at the species level between the control group and the group of mice receiving combined LPS + ZDV therapy. An increase in the abundance of the bacterial species *Muribaculaceae bacterium* (from 1.22% ± 0.42 to 2.17% ± 0.52, *p* < 0.05) was detected in the LPS + ZDV group compared to the control group (Figure 10 and Figure 11). In addition, we observed an increase in the abundance of species in the LPS + ZDV + *B. subtilis* group relative to the control: GGB34076 SGB48245 (family *Muribaculaceae*) (from 2.26% ± 1.39 to 5.41% ± 1.21, *p* < 0.05), GGB27849 SGB40283 (family *Muribaculaceae*) (from 0.51% ± 0.23 to 1.11% ± 0.18, *p* < 0.05), *Barnesiella* sp. WM24 (from 0.53% ± 0.36 to 1.72% ± 0.52, *p* < 0.05), GGB50207 SGB70279 (family *Muribaculaceae*) (from 0.66% ± 0.22 to 4.07% ± 1.32, *p* < 0.001) (Figure 10).

In contrast, in the LPS + ZDV + *Lactobacillus* spp. group compared to the control group, we observed a statistically significant decrease in the species: GGB23844 SGB35575 (phyla *Bacteroidota*) (1.22% ± 0.42 vs. 1.84% ± 0.34, *p* = 0.002), GGB28265 SGB40817 (family *Bacteroidaceae*) (0.93% ± 0.34 vs. 0.79% ± 0.33, *p* = 0.031). Meanwhile, the species GGB34076 SGB48245 (family *Muribaculaceae*) conversely increases in the LPS + ZDV + *Lactobacillus* spp. group relative to the control (from 2.27% ± 1.39 to 6.21% ± 1.50, *p* = 0.031) (Figure 11).

### 3.5. Effect of ZDV, LPS, and Probiotics on Histological Parameters of Mouse Intestine

**Figure 12:**
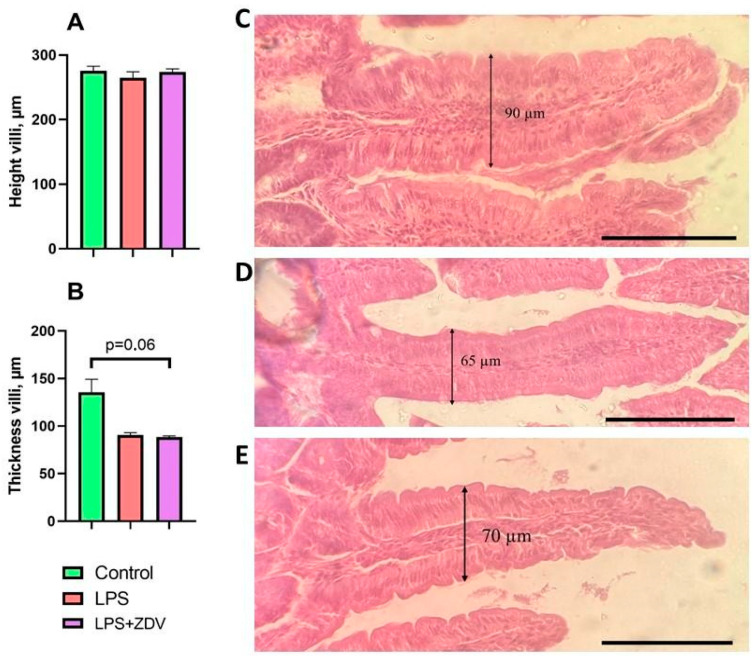
Effect of the studied substances on the length and thickness of intestinal villi. Villus length (**A**), villus thickness (**B**), representative examples showing villus thickness in mice from the control group (**C**), LPS group (**D**), LPS + ZDV groups (**E**). Semi-thin section. Stained with hematoxylin-eosin. Magnification 40×, scale 100 µm. Control, *n* = 12; LPS, *n* = 12; LPS + ZDV, *n* = 12.

The length and thickness of intestinal villi were assessed. None of the studied types of therapy directly affected villus length (Figure 12A), but mice receiving combined LPS + ZDV therapy showed a tendency towards a 35% decrease in villus thickness compared to the control; however, the differences were not statistically significant (*p* = 0.06) (Figure 12B–E). Administration of probiotic mixtures did not have a significant effect on intestinal villus dimensions.

**Figure 13:**
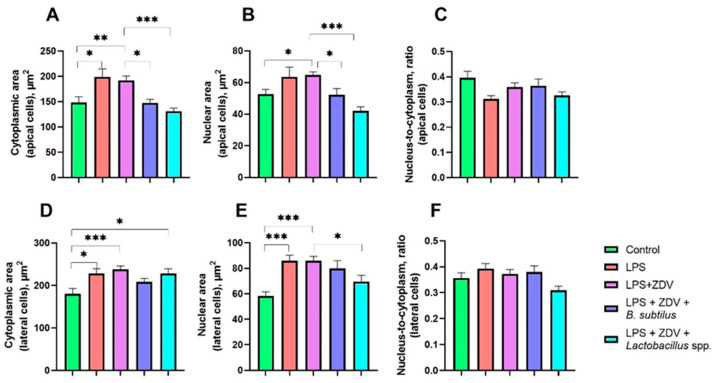
Effect of LPS and ZDV therapy, as well as probiotic intake, on parameters of apical cells (**A**–**C**) and lateral cells (**D**–**F**), such as cytoplasm area (**A**,**D**), nucleus area (**B**,**E**), nucleo-cytoplasmic ratio (**C**,**F**). Control, *n* = 12; LPS, *n* = 11; LPS + ZDV, *n* = 12; LPS + ZDV + *B. subtilis*, *n* = 12; LPS + ZDV + *Lactobacillus* spp., *n* = 12. Differences between experimental groups are statistically significant: * *p* < 0.05, ** *p* < 0.01, *** *p* < 0.001 (Kruskal–Wallis test, Dunn’s post hoc test).

An increase in the cytoplasm area in cells of the apical part of villi was observed in the group of mice receiving both LPS treatment alone (by 34%, *p* < 0.05) and combined LPS + ZDV therapy (by 30%, *p* < 0.01). At the same time, in mice receiving the *B. subtilis* mixture, the cytoplasm area of apical villi was 32% lower compared to mice from the LPS + ZDV group (*p* < 0.05), and in mice receiving the *Lactobacillus* spp. probiotic mixture was 48% lower (*p* < 0.001) (Figure 13A). Similarly, combined therapy contributed to an increase in the nuclear area of cells in the apical part of villi by 22% compared to the control. Probiotic intake prevented the increase in the nuclear area of these cells. This indicator was reduced in the LPS + ZDV + *B. subtilis* group by 25% (*p* < 0.05), and in the LPS + ZDV + *Lactobacillus* spp. group by 56% (*p* < 0.001) (Figure 13B). It should be noted that no statistically significant differences in the nucleo-cytoplasmic ratio in apical cells of intestinal villi were found between groups (Figure 13C).

Similar cell size patterns were observed in lateral villus cells. Cytoplasm area was increased in mice receiving LPS and the LPS + ZDV combination by 26% and 33%, respectively (*p* < 0.05 and *p* < 0.001, respectively). A similar increase of 21% was noted in the LPS + ZDV + *Lactobacillus* spp. group (*p* < 0.05), whereas in the LPS + ZDV + *B. subtilis* group no such increase was noted (Figure 13D). A significant increase in nucleus area was noted both in groups of mice receiving LPS injections (by 32%, *p* < 0.001) and combined LPS + ZDV therapy (by 32%, *p* < 0.001). At the same time, in the LPS + ZDV + *Lactobacillus* spp. group, a decrease in nucleus area relative to the LPS + ZDV group by 19% was observed (*p* < 0.05) (Figure 13E). This, apparently, was the reason why the LPS + ZDV + *Lactobacillus* spp. group showed the lowest nucleo-cytoplasmic ratio value (21% lower than in the group of mice receiving LPS injections, *p* < 0.01) (Figure 13F).

**Figure 14:**
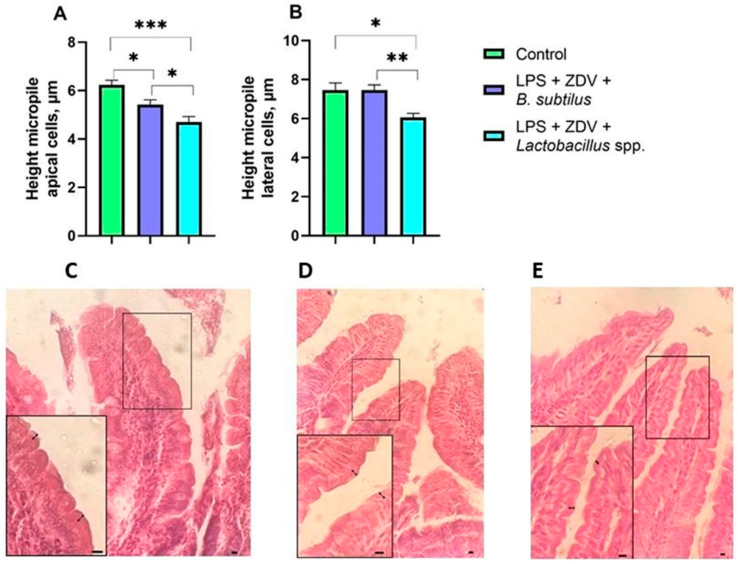
Effect of probiotics on the height of apical microvilli (**A**) and lateral microvilli (**B**). Representative examples showing the height of intestinal microvilli in mice from the control group (**C**), LPS + ZDV + *B. subtilis* group (**D**), LPS + ZDV + *Lactobacillus* spp. group (**E**). Semi-thin section. Stained with hematoxylin-eosin. Magnification 40×, scale 10 µm. The height of apical and lateral microvilli is marked by arrows. Control, *n* = 12; LPS + ZDV + *B. subtilis*, *n* = 12; LPS + ZDV + *Lactobacillus* spp., *n* = 12. Differences between experimental groups are statistically significant: * *p* < 0.05, ** *p* < 0.01, *** *p* < 0.001 (Kruskal–Wallis test, Dunn’s post hoc test).

Neither LPS therapy alone or combined LPS + ZDV therapy affected microvilli size. However, mice receiving the *Lactobacillus* spp. mixture showed the smallest microvilli height both in the apical part (Figure 14A) and in the lateral part (Figure 14B).

### 3.6. Correlations of Gut Bacterial Species with MWM Performance, Intestinal Morphometry, and Leukocyte Counts

To investigate potential relationships between the gut microbiota and the studied parameters, a Spearman correlation analysis with Bonferroni correction was performed. Several significant correlations were identified at a threshold of *p* < 0.01.

Analysis of the MWM performance revealed multiple associations with bacterial abundance. A positive correlation was found between the time spent searching for the platform on test day 6 and the bacterial species *GGB30484 SGB43516* (family *Oscillospiraceae*), *GGB27768 SGB40182* (phyla *Bacteroidota*), and *GGB28905 SGB41598* (family *Lachnospiraceae*) (r_s_ = 0.68, r_s_ = 0.73, and r_s_ = 0.68, respectively, all *p* < 0.01). Conversely, the time spent on test day 12 showed a negative correlation with the species *Erysipelotrichaceae bacterium* (family *Erysipelotrichaceae*) (r_s_ = −0.65, *p* < 0.01). The distance swam by the mice before locating the platform (path length) also exhibited significant correlations. On test day 6, the distance negatively correlated with *Phocaeicola vulgatus* (family *Bacteroidaceae*) (r_s_ = −0.64, *p* < 0.01) and *Akkermansia muciniphila* (family *Akkermansiaceae*) (r_s_ = −0.75, *p* < 0.001). A positive correlation on the same day was observed with *GGB27768 SGB40182* (phylum *Bacteroidota*) (r_s_ = 0.63, *p* < 0.01), *GGB28905 SGB41598* (family *Lachnospiraceae*) (r_s_ = 0.67, *p* < 0.01), and *GGB28835 SGB41494* (family *Lachnospiraceae*) (r_s_ = 0.62, *p* < 0.01). On test day 12, the distance correlated negatively with *GGB1379 SGB1880* (family *Bacteroidaceae*) (r_s_ = −0.67, *p* < 0.01), and positively with *Duncaniella muris* (family *Muribaculaceae*) (r_s_ = 0.62, *p* < 0.01), *Sangeribacter muris* (family *Muribaculaceae*) (r_s_ = 0.71, *p* < 0.01), and *GGB27914 SGB40352* (family *Muribaculaceae*) (r_s_ = 0.67, *p* < 0.01).

Regarding physiological and immunological parameters, only one bacterial species, *GGB27771 SGB40185* (family *Rikenellaceae*), demonstrated a negative correlation with intestinal length (r_s_ = −0.63, *p* < 0.01). A significant positive correlation with cf-mtDNA levels in blood was found exclusively for *GGB1575 SGB2164* (family *Bacteroidaceae*) (r_s_ = 0.61, *p* < 0.01).

Analysis of white blood cell populations revealed the following associations. The monocyte count correlated positively with *GGB27769 SGB40183* (family *Lachnospiraceae*) (r_s_ = 0.61, *p* < 0.01). The count of segmented neutrophils was negatively associated with *GGB27771 SGB40185* (family *Lachnospiraceae*) (r_s_ = −0.65, *p* < 0.01). Lymphocyte counts showed positive correlations with *GGB24127 SBG35930* (family *Oscillospiraceae*) (r_s_ = 0.57, *p* < 0.01) and *GGB28327 SBG40901* (family *Lachnospiraceae*) (r_s_ = 0.60, *p* < 0.01). Finally, eosinophil counts were positively correlated with *Levilactobacillus brevis* (family *Lactobacillaceae*) (r_s_ = 0.70, *p* < 0.001), *GGB29685 SBG42494* (family *Oscillospiraceae*) (r_s_ = 0.79, *p* < 0.001), *GGB81395 SBG63219* (family *Lachnospiraceae*) (r_s_ = 0.57, *p* < 0.01), and *GGB75880 SBG103680* (family *Oscillospiraceae*) (r_s_ = 0.65, *p* < 0.01).

## 4. Discussion

Our results show that combined exposure to LPS and ZDV causes a pro-inflammatory and toxic load on the entire organism. This combination led to significant body weight loss (Figure 2), a tendency towards hepatomegaly (Figure 3A), and pronounced impairments in spatial memory (Figure 4). This is consistent with the concept that the combined use of a pro-inflammatory agent, such as LPS, and a substance damaging the intestinal barrier, microbiome, and causing mitochondrial dysfunction creates a vicious cycle, enhancing systemic inflammation and endotoxemia. It has previously been shown that combinations of LPS with other agents, such as thrombin [17] and ovalbumin [16], can lead to more pronounced pathological changes compared to monotherapy. The fact that neither LPS nor complex LPS + ZDV therapy caused an increase in the amount of mtDNA damage in the brains of mice appears surprising (Figure 5A). It was previously shown that ZDV causes persistent mtDNA damage in newborn mice [18]. Presumably, in mature mice, the mitochondrial genome possesses greater resistance to antiretroviral drugs. Our gene expression analysis further supports this synergistic pro-inflammatory effect. The LPS + ZDV combination significantly upregulated *Tnfa* expression in the brain (*p* < 0.05), confirming the induction of neuroinflammation (Figure 5G).

It is important to note that the combined LPS + ZDV therapy caused persistent disturbances in the gut–brain axis. In particular, a persistent increase in the level of bacteria from the *Muribaculaceae* family was noted (Figure 9, Figure 10 and Figure 11). It is one of the common families in the mouse intestine [32]. It is known that this bacterial family is capable of producing short-chain fatty acids from both endogenous mucin glycans and exogenous polysaccharides [33]. Studies also show that representatives of this bacterial family are capable of degrading mucin [34]. However, it has previously been shown that an increase in the relative abundance of *Muribaculaceae* was associated with the development of acute pancreatitis in mice [35]. It has also been noted that this family may produce inflammatory processes in the organism [36]. According to the results of our study, a statistically significant increase in bacteria of the species GGB24127 SGB35930 (family *Muribaculaceae*) was obtained in the group already with LPS monotherapy relative to the control (Figure 9). A statistically significant increase in the abundance of *Muribaculaceae* bacterium was observed with combined therapy. Presumably, the increase may reflect a compensatory response of the microbiota to inflammation [33], aimed at enhancing enzymatic activity and attempting to restore homeostasis. It is also possible that, depending on the specific species, *Muribaculaceae* bacterium may be a pro-inflammatory factor [37]. We demonstrated that between the quantity of the bacterial species GGB1575_SGB2164 (family *Muribaculaceae*) and freely circulating mtDNA, a positive correlation was observed. In the study by Yang et al., it is shown that under the influence of dextran sulfate sodium, bacteria of this family are able to migrate from the intestine to the pancreas and cause local inflammation [38]. In the study by Mengozzi et al. on cardiac diseases, it is shown that the level of freely circulating mtDNA increases during inflammation [39]. In principle, it is currently believed that cf-mtDNA can act as a powerful damage-associated molecular pattern (DAMP), which has been most intensively studied in recent years [40]. Thus, we have reason to believe that changes in the level of bacteria from the *Muribaculaceae* family and the increase in the amount of mtDNA may be interrelated processes, showing the involvement of mitochondrial quality control in the regulation of the brain–gut axis.

We noted a decrease in the abundance of the species GGB23844 SGB35575 phylum *Bacteroidetes* upon induction of inflammatory processes compared to the control group (Figure 9). It was previously demonstrated that one of the most common bacterial phyla in mice was *Bacteroidetes*, and it was also noted that the proportion of *Bacteroidetes* decreased in obese animals compared to their lean counterparts [41]. It is also worth noting the fact that the abundance of *Bacteroidetes* contributes to the production of mucosal glycans, and a thick mucus layer can protect against damage to the colon [42]. Thus, this indicates the presence of inflammatory processes in the intestines of mice already even with LPS monotherapy.

Studies showing an association of the bacterial species *Sutterella wadsworthensis* with the gut microbiome of mice were not found in the literature analysis. However, research shows that the prevalence of *S. wadsworthensis* in the human gut microbiome has been associated with a number of diseases such as: multiple sclerosis, schizophrenia, migraine, and also obesity [43,44,45]. In turn, it is noted that a decrease in the abundance of representatives of this species was associated with remission in ulcerative colitis [46]. However, our obtained results regarding the abundance of this bacterial species in fecal samples from mice (Figure 9) are not consistent with the literature data. It can be concluded that insufficient research has been conducted assessing the influence of *S. wadsworthensis* on the host organism.

It was previously noted that the administration of *Phascolarctobacterium succinatutens* to mice contributed to a reduction in inflammatory processes in the intestine, as well as an increase in the amount of circulating arginine in the organism [47]. Nevertheless, a higher prevalence of *P. succinatutens* was demonstrated in overweight mice [48]. At the same time, this species positively correlated with the risk of developing multiple sclerosis and metabolic dysfunction-associated fatty liver disease, and was enriched in patients with sarcopenia [49]. In our study, the abundance of *P. succinatutens* in the LPS group increased relative to the control group (Figure 10), which may indicate the influence of representatives of this bacterial species on the production of inflammatory processes in the host organism.

Histological changes in the intestine also indirectly confirm damage to the intestinal barrier. Following combined therapy, we observed thinning of the villi (Figure 12), as well as an increase in the area of the cytoplasm and nucleus in apical and lateral enterocytes (Figure 13), which is characteristic of an inflammatory process [50]. Hydropic degeneration of the cytoplasm is accompanied by cell swelling [51,52]. And an increase in nuclear volume may indicate its preparation for lysis [53].

We believe that changes in the regulation of the brain-gut axis ultimately became one of the key causes of the development of cognitive deficits we observed in mice (Figure 4). We observed a strong negative correlation between the quantity of the bacterial species *Akkermansia muciniphila* (family *Akkermansiaceae*) and the distance mice swam on the 6th testing day (r_s_ = −0.75, *p* < 0.001). This indicates that a higher number of representatives of this species is associated with improved cognitive properties in mice. It has previously been shown that bacteria of this species contribute to the improvement of cognitive functions, as demonstrated in many studies [34,54,55]. The study by Khalili et al. on mice showed that bacteria of the species *Akkermansia muciniphila* positively affect the results of tests aimed at studying cognitive functions [51]. The study by Ahn et al. demonstrated an improvement in mouse memory through BDNF activation after feeding *Akkermansia muciniphila* [56]. Brain-derived neurotrophic factor (BDNF) is involved in neuroplasticity for the formation of new neural connections [57,58].

A strong negative correlation was also found between the quantity of the bacterial species *Phocaeicola vulgatus* (family *Bacteroidaceae*) and the average distance traveled during reverse learning in the Morris maze (r_s_ = −0.73, *p* < 0.001). However, these data rather contradict previously obtained results. Bacteria of this genus are capable of producing D-lactate [59], which serves as a metabolic fuel for neurons, necessary for improving brain function, enhancing memory, and influencing synaptic stability [60]. In the study by Cerdó et al., which involved assessing cognitive functions after transplanting bacteria of the genus *Phocaeicola* from newborn infants to mice, it was found that their high content was associated with improved cognitive functions [60]. On the other hand, it has been shown that *P. vulgatus* can cause septic shock [61], so it is clear that further study of the role of this microorganism species in the development of inflammatory reactions and its effect on cognitive processes is required.

We discovered a positive correlation between the content of the species *Levilactobacillus brevis* (family *Lactobacillaceae*) and GGB29685_SGB42494 (family *Eubacteriaceae*) and the content of eosinophils in the blood (r_s_ = +0.70, *p* < 0.001 and r_s_ = +0.79, *p* < 0.001, respectively). *Eosinophilia* is a characteristic feature of chronic inflammation [62]. Representatives of the *Eubacteriaceae* family have recently been discovered, whose genomes contain genes associated with the production of hydrogen sulfide, which poses a potential risk for disease development [60]. It was also previously shown that the abundance of bacteria of the genus *Anaerofustis* (family *Eubacteriaceae*) positively correlates with mortality in antibiotic-associated diarrhea [63], and a significant enrichment of bacteria of the genus *Anaerofustis* in the gut microbiota of patients with inflammatory bowel diseases was noted [64]. Although such data currently do not exist for the strain GGB29685_SGB42494, we can assume that its positive correlation with eosinophil levels may similarly indicate their potential pro-inflammatory properties.

A negative correlation was also found between the content of monocytes in the blood and the distance mice swam in search of the platform (r_s_ = −0.71, *p* < 0.001). So, it can be concluded that a higher level of monocytes in peripheral blood contributes to improved cognitive abilities. Although an increase in monocyte levels is usually associated with inflammatory reactions [65], it is known that some monocyte subpopulations play an important role in hippocampal neurogenesis [66], which may partially explain the positive correlation between monocyte levels in blood plasma and the cognitive functions of mice against the background of induced inflammatory processes.

Our data show that probiotic intake can mitigate the toxic effect of combined LPS + ZDV therapy, but there is a clear differentiation in the action of the two probiotic regimens. Comparative studies of the effects of *B. subtilis* and *Lacticaseibacillus rhamnosus* (until 2020 also classified in the genus *Lactobacillus*) have previously been conducted. It was shown that *B. subtilis* slowed the development of paralysis and extended the lifespan of *Caenorhabditis elegans* by activating mitophagy, which contributed to the removal of damaged mitochondria and thereby improved mitochondrial functions [67]. In our study, mice that received *B. subtilis* against the background of combined LPS + ZDV therapy also had increased expression of the *Pink1* gene in the brain (Figure 5C). In addition, we found that *B. subtilis* stimulated the expression of *Pten*, which, like *Pink1*, is a key regulator of mitophagy (Figure 5D). Mitophagy is a key process of mitochondrial quality control aimed at the selective autophagy of damaged or dysfunctional organelles. In the brain, maintaining effective mitophagy is critically important for neuronal health, as the accumulation of defective mitochondria is a source of excess oxidative stress, contributing to neurodegeneration and cognitive decline. The key role in initiating mitophagy is played by the PINK1/Parkin cascade. Upon loss of the mitochondrial membrane potential, the protein kinase *PINK1* stabilizes on the outer membrane of the damaged mitochondrion, which recruits and activates the E3-ubiquitin ligase Parkin. This leads to the polyubiquitination of outer membrane proteins, marking the mitochondrion for recognition by the autophagosome and subsequent degradation in lysosomes [68]. It is known that PTEN physically interacts with the E3-ubiquitin ligase Parkin, which is an important mediator of mitophagy [69]. Thus, activation of this pathway serves as a protective mechanism, cleansing the neuronal mitochondrial pool of damaged units, which is especially important under conditions of neuroinflammation caused by agents such as LPS. Furthermore, PTEN, as a key negative regulator of the PI3K/Akt/mTOR pathway, creates intracellular conditions favorable for the induction of autophagy [70]. Thus, the observed co-expression may reflect the activation of a coordinated network of stress responses aimed at enhancing mitochondrial quality control. Our data indirectly indicate that *B. subtilis* stimulates mitophagy through a *PINK1*- and *PTEN*-dependent mechanism.

It is also worth noting the positive changes that occurred in the gut microbiome. In the LPS + ZDV + *B. subtilis* group, the diversity of microorganisms was significantly increased (Figure 8A), which is a widely recognized indicator of the resilience and health of the gut ecosystem [71], as well as a factor influencing cognitive functions [66]. The genus *Barnesiella* is a representative of the phyla *Bacteroidota* and is associated with the ability to modulate the immune response and compete with opportunistic pathogens [72]. In our study, the highest abundance of *Barnesiella* sp. was found in the LPS + ZDV + *B. subtilis* group, indicating an enrichment of the microbiome with this taxon in response to *B. subtilis* administration. Species of the genus *Barnesiella* are often considered favorable commensals [36]. It has been shown that they possess the ability to produce isovaleric acid and other short-chain fatty acids (SCFAs) [73]. The anti-inflammatory effect of *B. subtilis* is further confirmed by the fact that no increase in the level of cf-mtDNA was observed in the blood plasma of mice (Figure 5E). Moreover, *B. subtilis* intake reduced the level of *Fis1* gene expression in the brain (Figure 5B). It is known that overexpression of *Fis1* leads to increased mitochondrial division and fragmentation, which is observed in various acute and chronic neurological disorders, including stroke, traumatic brain injury, Alzheimer’s disease, and Parkinson’s disease [74]. Consistently, in our experiment, mice from the LPS + ZDV + *B. subtilis* group did not exhibit such pronounced cognitive deficits as noted in mice receiving combined LPS + ZDV therapy (Figure 4). Although *Il1b* and *Il6* showed only a non-significant trend toward increase in the LPS + ZDV group, their expression was significantly reduced in mice receiving *B. subtilis* compared to the LPS + ZDV group (both *p* < 0.05) (Figure 5H,I). This suggests that while the combined therapy triggers a neuroinflammatory response, *B. subtilis* exerts a broad anti-inflammatory effect by suppressing key pro-inflammatory cytokines.

Ambiguous results were obtained for the LPS + ZDV + *Lactobacillus* spp. group. Contrary to expectations, the lactobacilli mixture not only did not improve but in some aspects exacerbated the consequences of the combined therapy. According to some indicators, cognitive dysfunctions in mice from the LPS + ZDV + *Lactobacillus* spp. group was even more pronounced than in mice in the LPS + ZDV group (Figure 4). However, we can note a 5-fold decrease in monocyte levels compared to the LPS group (Figure 5F). Although this may indicate a powerful anti-inflammatory action, in this context it more likely points to excessive immunosuppression or disruption of immune cell migration, which prevented an adequate response to the damage. The anti-inflammatory properties of lactobacilli, including modulation of the nuclear factor kappa-light-chain-enhancer of activated B cells (NF-κB) pathway and reduction in interleukin-6 (*IL-6*) production, are well-documented [75]. However, the concept of strain-specificity and context-dependent action of probiotics is critically important. Some *Lactobacillus strains* (in particular *L. plantarum*) in certain inflammatory models did not provide a protective effect and, on the contrary, even enhanced the inflammatory response [76]. Our obtained data may indicate that the combination of strains *L. helveticus*, *L. plantarum*, *L. paracasei* under conditions of combined LPS and antiretroviral drug toxicity could exhibit undesirable immunomodulatory properties. An important morphological confirmation of this proved to be insufficiently effective specifically in this model and under these experimental conditions is the observed significant shortening of intestinal microvilli in this group (Figure 14). Small intestine microvilli play an important role in the absorption of nutrients and trace elements necessary for the organism. Their shortening disrupts the functional work of the intestine, leading to insufficient intake of beneficial substances [77]. Thus, the chosen lactobacilli mixture likely proved unable to compensate for the severe intestinal damage caused by LPS and ZDV, and possibly even disrupted the processes of mucosal repair, which was reflected in the worsening of many functional outcomes.

Although our data suggest that *B. subtilis* exerts beneficial effects through the activation of the PINK1/PTEN-dependent mitophagy pathway in the brain, the mechanistic evidence presented is primarily correlational. While the observed changes in gene or protein expression support this hypothesis, further studies employing specific pathway inhibitors (e.g., PINK1 knockdown) or additional protein-level validation are required to establish a definitive causal link between the probiotic treatment, enhanced mitophagy, and the resulting neuroprotective effects.

## 5. Conclusions

This study demonstrates that combined exposure to LPS and ZDV induces significant dysfunction along the gut–brain axis, characterized by neuroinflammation, spatial memory impairment, intestinal damage, and dysbiosis with an expansion of pro-inflammatory *Muribaculaceae*. The administration of *B. subtilis* effectively counteracted these deleterious effects by activating PINK1/PTEN-dependent mitophagy in the brain, suppressing the expression of key pro-inflammatory cytokines, and restoring gut microbial diversity. In contrast, a mixture of lactobacilli failed to provide cognitive protection and was associated with exacerbated intestinal pathology, underscoring the critical importance of strain-specificity in probiotic interventions. These findings highlight *B. subtilis* as a promising candidate for mitigating cognitive impairments associated with gut–brain axis dysfunction and warrant further investigation into its therapeutic potential.

## Figures and Tables

**Table 1:** Starting positions in the Morris water maze.

Acquisition Learning	Start Point			
Day	Trial 1	Trial 2	Trial 3	Trial 4
1	N	E	SE	NW
2	SE	N	NW	E
3	NW	SE	E	N
4	E	NW	N	SE
5	N	SE	E	NW
6 (Test attempt)	NE			
Reversal learning				
1	S	W	NW	SE
2	NW	S	SE	W
3	SE	NW	W	S
4	W	SE	S	NW
5	S	NW	W	SE
6 (Reversal test attempt)	SW			

N—North, E—East, S—South, W—West.

**Table 2:** Primer sequences for measuring mtDNA damage.

Fragment	Forward Primer Sequence 5′→3′	Reverse Primer Sequence 5′→3′
1 long	TAAATTTCGTGCCAGCCACC	ATGCTACCTTTGCACGGTCA
2 long	ACGAGGGTCCAACTGTCTCTTA	CCGGCTGCGTATTCTACGTT
6 long	AAGAAGGAGCTACTCCCCACC	GTTGACACGTTTTACGCCGA
MitoShort	ACGAGGGTCCAACTGTCTCTTA	AGCTCCATAGGGTCTTCTCGT
NuclShort (Gapdh)	GGCTCCCTAGGCCCCTCCTG	TCCCAACTCGGCCCCCAACA

**Table 3:** Primer sequences for the assessment of gene expression.

Gene	Forward Primer Sequence 5′→3′	Reverse Primer Sequence 5′→3′
*Gapdh*	GGCTCCCTAGGCCCCTCCTG	TCCCAACTCGGCCCCCAACA
*Pten*	AGGGACGAACTGGTGTAATGA	GGGAATAGTTACTCCCTTTTTGTCT
*Pink1*	GAGCAGACTCCCAGTTCTCG	GTCCCACTCCACAAGGATGT
*Fis*	CTACAGGGGTGCAGGAGAAA	AGATGGACTGGTAGGCATGG
*Tnf*	TATGGCTCAGGGTCCAACTC	GGAAAGCCCATTTGAGTCCT
*Il1b*	TTGACGGACCCCAAAAGATG	AGAAGGTGCTCATGTCCTCA
*Il6*	CGGAGAGGAGACTTCACAGAG	CATTTCCACGATTTCCCAGA

## Data Availability

The original data presented in the study are openly available in the NCBI BioProject database (BioProject ID: PRJNA1392634).

## Abbreviations

LPS	Lipopolysaccharide
ZDV	Zidovudine
HLA	Human leukocyte antigen
TLR4	Toll-like receptor 4
mtDNA	Mitochondrial DNA
PCR	Polymerase chain reaction
DNBs	DNA-nanoballs
